# Synthesis and Characterization of a One-Dimensional Malleable Spin-Crossover Polymer Complex Modified by Methoxy Polyethylene Glycol

**DOI:** 10.3390/polym15102363

**Published:** 2023-05-18

**Authors:** Di Wang, Ren Zhang, Jin Liu, Bibi Ji, Wenping Wang, Mengyuan Peng, Chen Huang, Lizhuoran Cheng, Yi Ding

**Affiliations:** 1 School of Materials and Chemical Engineering, Anhui Jianzhu University, Hefei 230601, China; 2 School of Chemistry and Chemical Engineering, Hefei University of Technology, Hefei 230009, China

**Keywords:** spin crossover (SCO) complex, malleable, magnetic transition polymer, modification, MPEG films

## Abstract

A novel one-dimensional malleable spin-crossover (SCO) complex {[Fe(MPEG-trz)_3_](BF_4_)_2_} has been successfully synthesized by molecular self-assembly between 4-amino-1,2,4-triazoles (MPEG-trz) grafted with a long flexible chain methoxy polyethylene glycol (MPEG) and metallic complex Fe(BF_4_)_2_•6H_2_O. The detailed structure information was illustrated by using FT-IR and ^1^H NMR measurements, while the physical behaviors of the malleable SCO complexes were systematically investigated by using magnetic susceptibility measurements using superconductivity quantum interference device (SQUID) and differential scanning calorimetry (DSC). This new metallopolymer exhibits a remarkable spin crossover transition behavior, between two spin quantum states (Fe^2+^ ions): high spin (HS) state (quintet state) and low spin (LS) state (singlet state), at a specific critical temperature with a slender hysteresis loop of 1 K. DFT computations revealed the partial rules of HOMO-LUMO energy levels and spin density distributions of different four-position substituted [Fe(1,2,4-triazole)_3_]^2+^ derivatives with different length of repeat units in polymer complexes. This can go a step further to depict the spin and magnetic transition behaviors of SCO polymer complexes. Furthermore, the coordination polymers possess an excellent processability due to an outstanding malleability, which can be easily shaped into a polymer film with spin magnetic switching properties.

## 1. Introduction

Spin crossover (SCO) complexes, which are interesting spin dual stable-state systems containing 3d^4–7^ metal elements [1,2,3,4], feature spin transition behavior between the low spin state (LS, singlet state) and the high spin state (HS, quintuplet state) under relatively minor change of external stimuli factors such as temperature, light, and pressure [5,6,7,8,9,10]. Particularly, the iron (II) complexes with 3d^6^ outer shell of electron configurations and coordinated by triazole molecules are especially important, due to their broad thermal hysteresis loops around room temperature; swift and reversible transition and the stability of each quantum spin states [11] are also the essential factors [12] for potential applications as molecular sensors, molecular switches, data storage, and other electronic or quantum devices [13].

Up until now, a large number of iron (II) SCO coordination compounds (mono-, bi-, and multi-nuclear) have been reported as single crystal or powder or diluted materials [14,15]. However, a key obstacle for utilizing them is their poor processability. It is well known that a polymer could be easily designed and constructed into specific structures and morphologies as demanded according to their admirable processability and ductility. Thus, introducing flexible polymer chains into the SCO system by chemically modified methods could provide a promising strategy toward new malleable SCO materials [16,17,18,19].

**Scheme 1:**
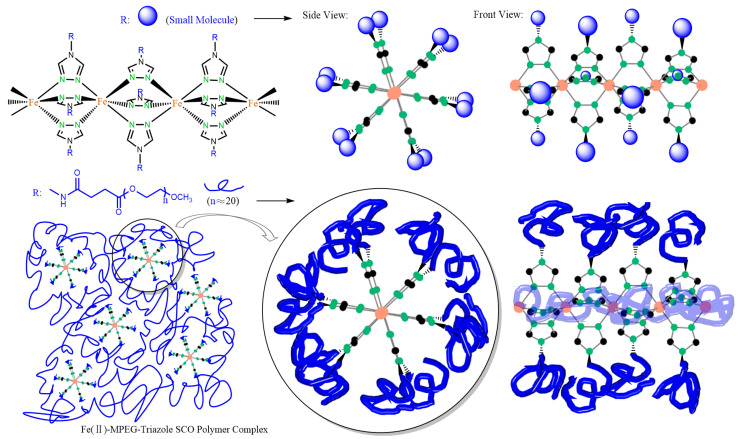
The schematic diagram of two kinds of SCO complexes, modified by small ligand and polymer ligand, respectively, from side view and front view.

M. Rubio et al. developed a gel phase ferrous ion complex with octadecyl chains, which not only possessed SCO properties but also exhibited gelation behavior [20]. In addition, J. A. Kitchen et al. attached hexadecyl group into SCO molecules to achieve a malleable spin crossover materials. However, the mechanical properties, especially the malleability was not provided in the article [21]. Meanwhile, according to these previous researches [22,23,24,25], the additional functional groups that modified the organic ligands may weaken the SCO behavior, due to the conversion of the intermolecular interactions between SCO chains, which play an important role in the transition of spin quantum state (HS ~ LS) along with hysteresis loop [26]. In order to solve this problem, R.J. Davidson et al. introduced a polyphosphazene structure repeat unit to the system to acquire malleable SCO materials. This ordered repeat unit is similar to cyclotriphosphazene derivatives. Possibly, the special structure had as small an impact as possible on intermolecular interactions [27]. Unfortunately, the rigid structure cyclotriphosphazene derivatives hindered the possibility of processability, but it provided us a probable or even feasible method to keep whole balance between processability and spin crossover property, when macromolecular substituents were attached to the ligand pendants. Scheme 1 shows the SCO Fe (II) triazole structures decorated with small molecular organic ligands (e.g., triazoles or triazole derivatives represented by small rigid balls) and flexible polymer chains, which is represented by flexible random coils. The diagram depicts the two kinds of triazole ligand structures coordinated with Fe (II) spin centers from side view and front view. The flexible random-coil-like structures enwrap the Fe (II)-triazole spin central chains and endow the whole SCO complex with more excellent processability.

**Scheme 2:**
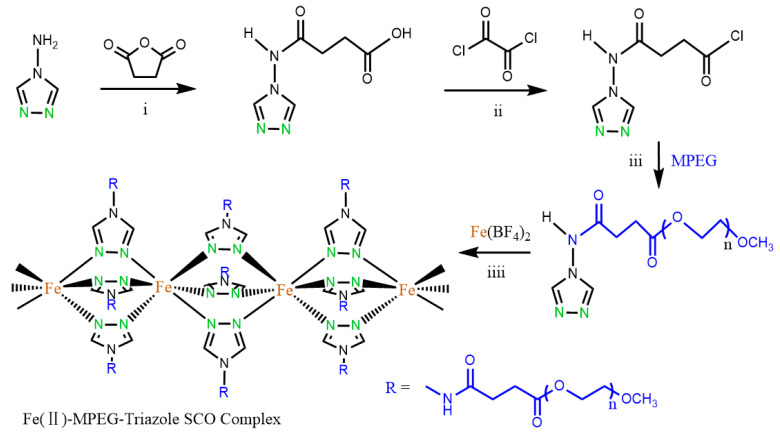
The synthesis schematic route of polymeric ligand from left top: (i) TEA, acetonitrile, 40 °C, 6.5 h; (ii) DCM, 0 °C, 6.5 h; (iii) TEA, DCM/DMF, rt, 12 h. The final designed structural formula of SCO polymer complex in left bottom. (iiii) Fe(BF_4_)_2_, methanol/acetone, rt, 30 min.

In our previous work [28], the PGMA (poly(glycidyl methacrylate))-decorated triazole iron (II) complex was synthesized by the combination of atom transfer radical polymerization (ATRP) and ring-opening reaction, which displayed not only a prominent spin crossover performance but also an excellent processability. As a continuous effort, we herein introduced the methoxy polyethylene glycol (MPEG) chains [29,30] to the organic ligand triazoles to give a flexible long chain structure feature with the repeating and regular units, ether bonds (MPEG-750, about 15~20 repeated units of polyethylene glycol, PEG) to prepare the SCO materials with an excellent processability so they could be further processed into a polymer thin film with an outstanding spin magnetic transition feature (the spin state/multiplicity convert between HS quintuplet to LS singlet). In the present work, we synthesized carboxyl-terminated 4-amino-1,2,4-triazole ligand by esterification reaction, which is prepared by amidation between 4-amino-1,2,4-triazole and succinic anhydride. Successively, upon the reaction of oxalyl chloride and the prepared MPEG-functionalized polymer chains finally afforded the target triazole-based ligands. The whole synthetic routes are summarized in Scheme 2. The characteristics of ^1^H NMR, FT-IR demonstrated the covalent linkage of the flexible polymer chain and Fe (II) triazole SCO materials. In order to investigate the relationship between the molecular structure induced by the MPEG long chain decorated on triazole ligand and the whole SCO properties, four different structures of the series of triazole-Fe (II) SCO coordination molecules, named complex **1**, **2**, **3,** and **4** were synthesized. They were designed and performed by the different proportions of MPEG-trz in total triazoles: 100%, 67%, 50%, and 33%, respectively. Superconductivity quantum interference device (SQUID) measurements revealed the spin magnetic properties in the range of temperature from 10 K to 300 K of the spin crossover performance of SCO materials functionalized by the MPEG chain, and differential scanning calorimetry (DSC) measurements also provided strong evidence for reversible spin transitions of the Fe (II) polymer complex; meanwhile, the crystal structures of the new SCO complex was characterized by using X-ray diffraction (XRD). In addition, DFT Gaussian computations was used in SCO polymer complexes for the first time and was reported to reveal the internal mechanism or the relationship between the molecular chain structure and the spin magnetic properties [31]. Because of the inappropriate of DFT computations for extremely big or long flexible polymer complexes due to the too many possible conformations, we made an approximation of different four-position substituted [Fe(1,2,4-triazole)_3_]^2+^ derivatives with different length of repeat units in SCO molecules and solved these computational problems above. The SCO rules of HOMO-LUMO energy levels and spin density distributions for the polymer complexes, which are affected by the substituted groups of ligand triazoles, and the coordination length of the molecular backbone were analogized properly by DFT simulations of derivatives structural approximation. We can partially foresee the spin magnetic properties of the longer Fe (II) polymer complex with big and flexible triazole derivative ligands through some concluded trends of calculation results.

## 2. Experimental

### 2.1. Materials and Characterizations

4-Amino-1,2,4-triazole (Aladdin, Wuhan, China) and succinic anhydride (Aladdin, China) were purified and recrystallized from acetic anhydride and acetonitrile two times, respectively, followed by washing with diethyl ether and finally, dried in a vacuum. Methoxy polyethylene glycol (Mn = 1K) denoted as MPEG 1K (Fluka, St. Louis, MO, USA) was anhydrated and dried by azeotropic distillation in toluene. Fe(BF_4_)_2_▪6H_2_O (Aladdin, China) and oxalyl chloride (Aladdin, China) are commercial. Trimethylamine (TEA), dichloromethane (DCM), *N*,*N*-dimethyl-formamide (DMF), and acetonitrile were anhydrated by stirring 24 h with CaH_2_ and distilled under reduced pressure before use. Other reagents mentioned in the synthetic route are commercial from Sinopharm Chemical Regent Co., Ltd. (Shanghai, China) and were directly used without further purification.

Fourier transform infrared (FT-IR) data were collected on a Bruker VECTOR-22 FT-IR spectrometer, by using KBr pellet with spectroscopic grade at rt. The ^1^H NMR spectra were recorded on a Bruker AVAN600 CE NMR instrument, by using D_2_O and tetramethylsilane (TMS) as solvent and internal reference, respectively. The characterization of magnetic properties were performed on a superconducting quantum interference device (SQUID) magnetometer, which is from Quantum Design (4 K ≤ T ≤ 300 K, ZFC, H ≤ 50 kOe) with an external field of 1 T. The differential scanning calorimetry (DSC) thermal measurements were performed on Mettler DSC 822 calorimeter in N_2_ atmosphere within the temperature range of −80 °C to 0 °C at a heating rate of 5 K/min. XRD spectra measurements were collected on a M18XHF-SPA X-ray diffraction instrument of Mac Science Co. with Cu Kα radiation (k = 1.5406 Å) at the scanning rate of 0.1 degree per second between 2*θ* = 5°–50°.

### 2.2. Synthesis of Carboxyl-Terminated 4-amino-1,2,4-triazole (NH_2_-trz-COOH)

4-Amino-1,2,4-triazole (0.504 g, 6 mmol), succinic anhydride (0.900 g, 9 mmol), and TEA (0.600 g, 6 mmol) were dissolved in anhydrous acetonitrile (80 mL); the reaction was performed at 40 °C for 6.5 h under vigorous stirring. Then, the solvent acetonitrile was evaporated by using a rotary evaporator and the residue was precipitated in ethyl acetate. The resulting solid was collected by filtration and dried at 40 °C in vacuum to afford NH_2_-trz-COOH in 73% yield.

^1^H NMR (600 MHz, D_2_O): *δ* (ppm) 8.56 (s, 1H, N=C-H), 2.74 (m, 4H, -COCH_2_CH_2_CO-); FT-IR (KBr, cm^−1^): 3123 cm^−1^–3310 cm^−1^, (-NH, stretching), 1538 cm^−1^ (C=N, stretching), 1713 cm^−1^ (C=O, stretching), 1670 cm^−1^ (-CONH-, stretching).

### 2.3. Synthesis of the Polymer-Modified Ligand MPEG-trz

A mixture *N*-trz-COOH (1.840 g, 10 mmol), oxalyl chloride (10 mL), DMF, and DCM (50 mL) were refluxed at 0 °C for 6.5 h under N_2_ atmosphere. The excessive oxalyl chloride was evaporated by using an evaporator. Subsequently, the residue was dissolved in 20 mL DMF and was dissolved into a mixture of MPEG (12.000 g, 12 mmol), DCM (40 mL), and TEA (1.200 g, 12 mmol); the reaction was carried out at rt for 12 h under N_2_ atmosphere. The reaction byproduct triethylamine hydrochloride was then removed by filtration, and the filtrate was evaporated totally. The resulting product was dissolved in chloroform, washed with diluted HCl solution (pH is about 5.0) and dehydrated over anhydrous Na_2_SO_4_. The crude product was purified by precipitating in diethyl ether. MPEG-trz was obtained in 85% yield.

^1^H NMR (600 MHz, D_2_O): *δ* (ppm) 8.50 (s, 1H, -N=C-H), 4.26 (t, 2H, -COOCH_2_), 3.68 (m, 4H, -OCH_2_CH_2_O-), 3.37 (s, 3H, CH_3_O-), 2.67 (m, 4H, -COCH_2_CH_2_CO-); FT-IR (KBr, cm^−1^): 3123 cm^−1^–3310 cm^−1^, (-NH, stretching), 1563 cm^−1^ (C=N, stretching), 1735 cm^−1^ (-COO-, stretching), 1650 cm^−1^ (-CONH-, stretching).

### 2.4. Synthesis of Fe (II) Complex via MPEG-trz and Fe(BF_4_)_2_•6H_2_O

Fe(BF_4_)_2_•6H_2_O (0.6750 g, 2 mmol), antioxidants ascorbic acid, methanol (10 mL), and acetone (10 mL) were added into a bottom of flask, then the solution (10 mL of methanol and 10 mL of acetone) of MPEG-trz (0.7000 g, 6 mmol) was slowly dropped in 20 min. After reacting for about 30 min, the white precipitate was formed at the bottom of the flask, then the product was dealt with the filtration, washed with methanol thoroughly and dried at 40 °C in vacuum. The Fe (II) complexes was obtained in the yield of 85%.

FT-IR (KBr, cm^−1^): 3418 cm^−1^ (-NH_2_), 1735 cm^−1^ (C=O, stretching), 1558 cm^−1^ (C=N, stretching).

## 3. Results and Discussions

### 3.1. Synthesis and Characterizations of Polymer Complexes

**Figure 1:**
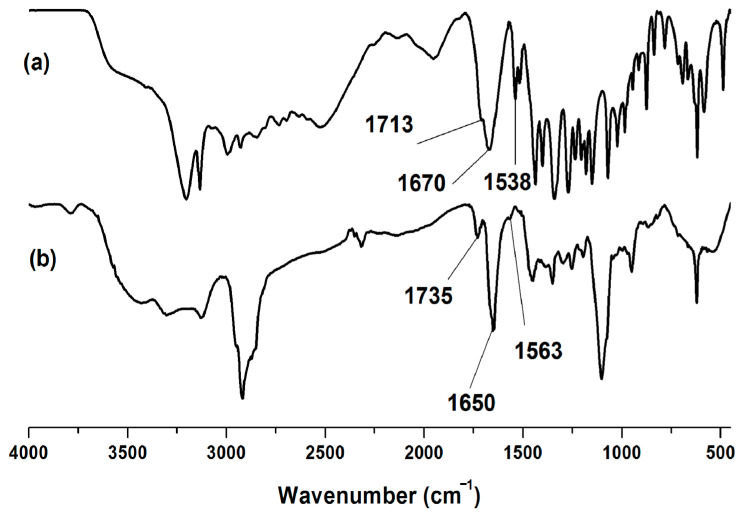
(**a**) FT-IR spectra of carboxyl-terminated triazole NH_2_-trz-COOH, (**b**) polymer-based MPEG-trz ligand.

**Figure 2:**
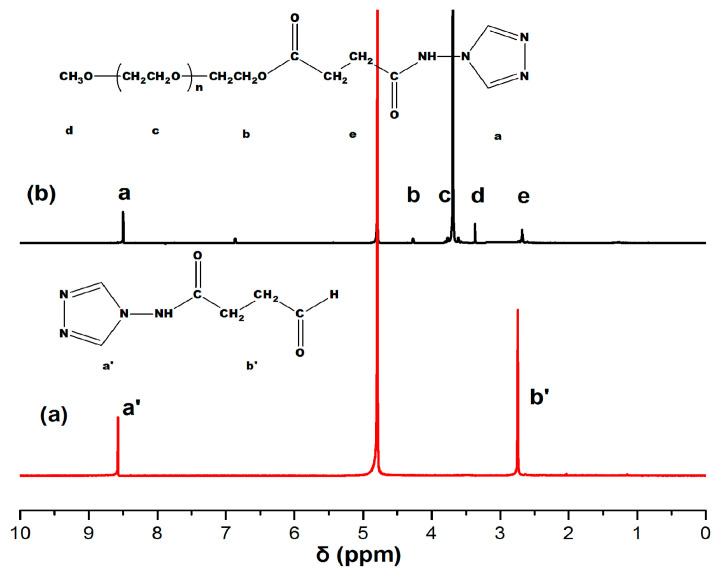
(**a**) ^1^H NMR spectra of carboxyl-terminated triazole NH_2_-trz-COOH, (**b**) polymer-based MPEG-trz ligand.

Preparation of the polymer-based triazole ligands (MPEG-trz). The NH_2_-trz-COOH used in the esterification with MPEG-OH were prepared by amidation of 4-amino-1,2,4-triazole ligand with succinic anhydride. The product was fully characterized using FT-IR and ^1^H NMR spectroscopy, which are shown in Figure 1 and Figure 2, respectively. The absorption band of amide carbonyl attributed at υ = 1670 cm^−1^, acid carbonyl for υ = 1713 cm^−1^, and carbon–nitrogen double bond in triazole ring for υ = 1538 cm^−1^ are shown in Figure 1a, combined with the chemical shift (-CH=N-) for triazole ring at *δ* = 8.56 ppm, -COCH_2_CH_2_CO- at *δ* = 2.74 ppm, which are shown in Figure 2a. These characteristic absorption bands indicating that NH_2_-trz-COOH has been successfully synthesized. The esterification of NH_2_-trz-COCl with a slight excess of reactant MPEG was carried out at rt in the presence of TEA. The absorption peaks at υ = 1735 cm^−1^, 1650 cm^−1^, and 1563 cm^−1^ attributed to ester carbonyl, amide carbonyl, and carbon–nitrogen double bond in triazole ring, respectively, shown in Figure 1b, combined with the new signals at *δ* = 4.26, 3.68, and 3.37 ppm assigned to –COOCH_2_-, -OCH_2_CH_2_O-, and CH_3_O- observed in Figure 2b, illustrate that the MPEG-trz has been successfully synthesized.

### 3.2. Characterizations and Description of Spin Polymer Complexes Films

**Figure 3:**
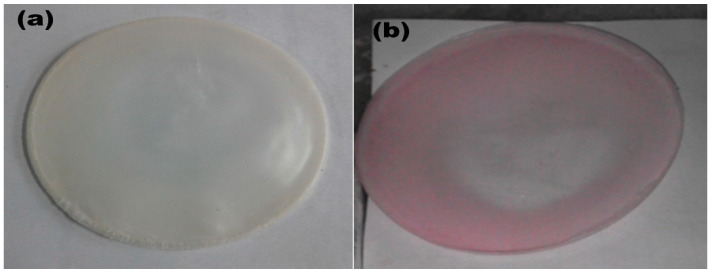
The exhibition of the thermochromism of flexible SCO polymeric film due to the spin transition phenomenon for Fe (II) coordination: (**a**) apparent white color in the form of film at the LS state (T = 300 K) and (**b**) apparent purple in the film at the LS state (T = 77 K).

According to previous reports, the SCO behavior could be directly triggered by external stimuli such as temperature, pressure, magnetic field, or light irradiation; meanwhile the spin transition process is always accompanied with color change: white is HS and purple is LS. Consequently, we can estimate whether the spin transition happens or not, just qualitatively according to the color change of the coordination complex under different temperatures. Furthermore, to characterize its mechanical behavior, especially the malleability, we shaped the newly prepared MPEG chain-functionalized SCO complex into a polymeric film for about 40 μm thickness at room temperature by the solution salivate method. Firstly, 5% (mass) DCM solution of poly (lactic acid) (PLA) was prepared, and the compounds were evenly dispersed inside. After the evaporation, the SCO polymeric film was prepared. We can clearly observe that the SCO polymeric film is white at rt (the film object is shown in Figure 3a, which actually shows that the spin quantum state of the material stays in the high spin state), while its color becomes purple as T decreases to 77 K, shown in Figure 3b, which is the energy characteristic of d-d electronic transitions of LS Fe (II) species. The thermochromism directly indicates that the MPEG-modified Fe (II) complex still retains the spin-state transition property derived from the original SCO Fe (II) complex.

### 3.3. Magnetic Properties

**Figure 4:**
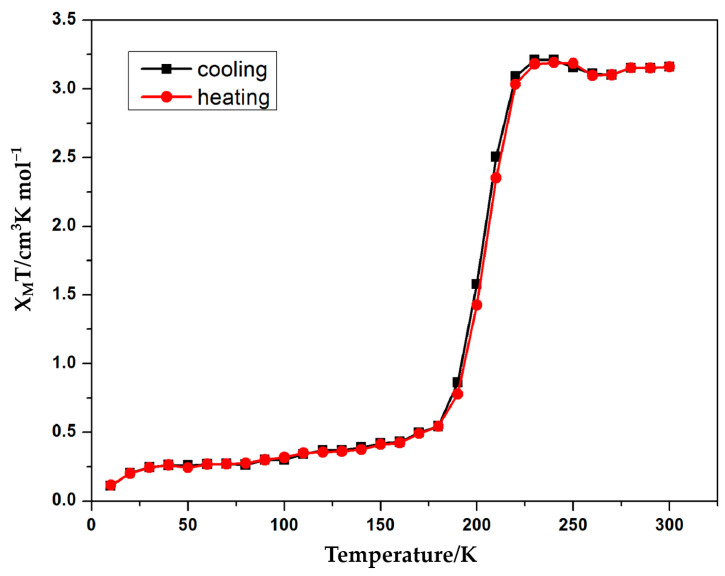
The magnetic susceptibility χ multiply by T versus T profile of the SCO coordinations. The T changing modes of cooling and heating processes are in black and red, respectively.

In order to quantitatively test the SCO behavior of our products, we applied the SQUID to record the χ_M_T of the newly prepared Fe (II)-MPEG-modified complexes with the temperature increase or decrease in the whole system (the range is from 10 K to 300 K, in external field 1 T). The results of magnetic properties and spin transition behaviors are presented in Figure 4. A notable and swift increase can be observed in molar magnetic susceptibility χ_M_T nearly ten times, up to 3.2 cm^3^ K mol^−1^ from around 0.3 cm^3^ K mol^−1^ at the temperature range of 180 K and 220 K. The clear and quick increase in χ_M_T exhibit a reliable spin transition from LS to HS. The transitions in both directions which are from LS to HS or from HS to LS, respectively, are clear and complete. The T_c_↓ and T_c_↑ are 205 K and 206 K (the critical temperature, at which one-half of the SCO behavior is completed during cooling process and heating process, respectively), which means the width of magnetic hysteresis loop is approximately 1 K. In fact, the spin magnetic properties, T_c_ of transitions or the width of magnetic hysteresis loop mainly depend on the coordinated environments around the Fe^2+^ ion centers. The χ_M_T value for the low-spin state of Fe (II) species was not zero before the SCO critical point. The reason is the χ_M_T values of coordinated polymers of LS species most possibly originate from the steric hindering effect of pendants polymers coordinated to Fe (II) spin center ions. This steric effect always leads the complex more likely to adopt a high spin state and be more easily hydrated. So, the polymer pendants on triazole ligands would hinder the chain length of Fe (II) ions and triazole coordination complexes. Therefore, the introduction of MPEG exerts a huge impact on spin crossover system for molar magnetic susceptibility and the critical temperature of spin transition T_c_.

**Figure 5:**
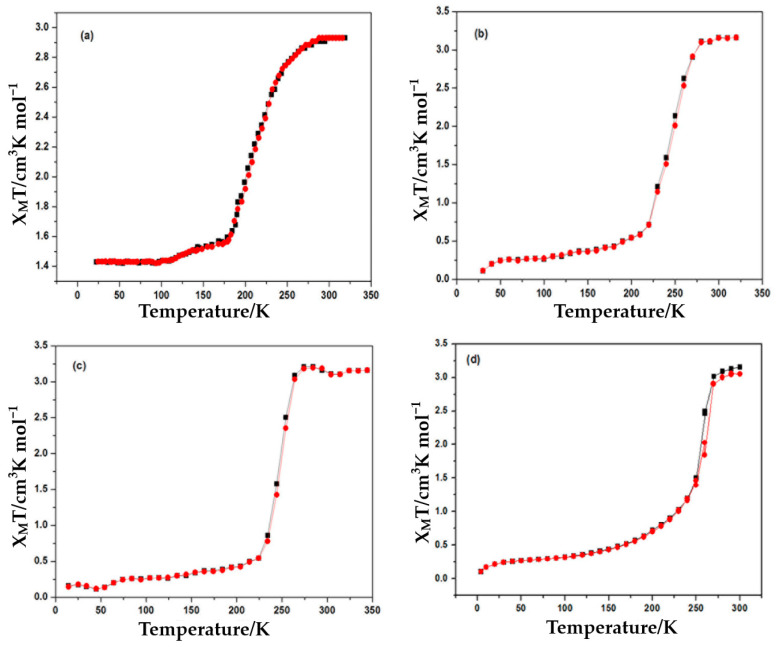
The magnetic susceptibility χ multiplied by T versus T profile of the SCO coordination complexes. Complex **1** for (**a**), **2** for (**b**), **3** for (**c**), **4** for (**d**), respectively. The T changing modes of cooling and heating processes are in black and red, respectively.

For the sake of investigating the influences of the different ratios between MPEG-trz and NH2-trz ligands introduced into the complexes on spin magnetic properties, we synthesized a series of triazole- Fe (II) SCO materials with the proportions of MPEG-trz in total triazoles: 100%, 67%, 50%, 33%, named complex **1**, **2**, **3**, **4**, respectively, by using the same method with the above SCO complex. The molar magnetic susceptibility χ_M_T of the four complexes, before or after the spin transition belonging to LS and HS, respectively, were summarized in Figure 5. In addition, The T_c_↓ and T_c_↑ of spin transition for complex **1**, **2**, **3**, **4** are shown in Table 1. Complex **1** has the same ratio with the former independent SQUID sample. The T_c_↓ and T_c_↑of spin transition and the deviation between them are nearly the same.

### 3.4. Spin Crossover Properties by Polymer Thermal Analysis

**Figure 6:**
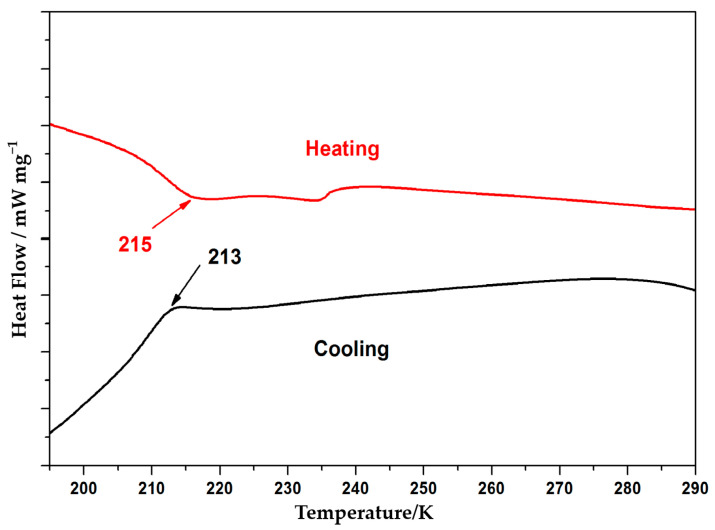
DSC spectrum of spin crossover complex. The two curves at top and bottom represent cooling and heating modes, respectively.

For the sake of studying the spin crossover behavior step further, differential scanning calorimetry (DSC) was applied to study the thermal properties of the newly synthesized SCO complex by monitoring the enthalpy change ∆H during the heating and cooling procedures. Although at the range of 195~290 K (shown in Figure 6), the MPEG-modified Fe (II) complex did not display an abrupt and drastic endothermic/exothermic curve, an obvious change of slope or turning point was observed during the cooling or heating process. The critical turning point of temperature T_exot_ was 213 K, and T_endo_ was 215 K, which refer to exothermic and endothermic process, respectively. From the data of DSC curves, we can conclude that the phase change of the spin transition process is a typical second-order phase transition process. Considering the SQUID measurement results, it is also confirmed in another angle that the spin crossover phenomenon existed and occurred between HS and LS.

**Figure 7:**
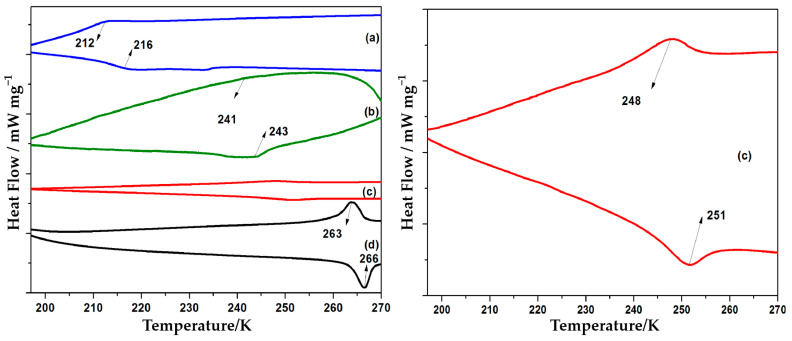
DSC spectra of the SCO coordination complex. Complex **1** for (**a**), **2** for (**b**), **3** for (**c**), **4** for (**d**), respectively. The two curves for each complex at top and bottom represent cooling and heating modes, respectively. The right picture is the amplified plot of complex **3**.

In addition, we also measured the DSC of SCO materials complex **1**, **2**, **3**, **4**, which were synthesized by the same proportion of two triazoles (MPEG-trz and NH2-trz ligands) used in SQUID measurements mentioned above. The thermal property curves in exothermic and endothermic process of the four complexes are shown in Figure 7a–d, respectively, below. The critical turning point of temperature T_exot_ and T_endo_ (the point at which the slope changes) for each of the four complexes are shown in Table 2. Complex **1** has the same ratio with the former independent DSC sample. The T_exot_ and T_endo_, which are also represented the points of spin magnetic transition, are approximately the same.

**Figure 8:**
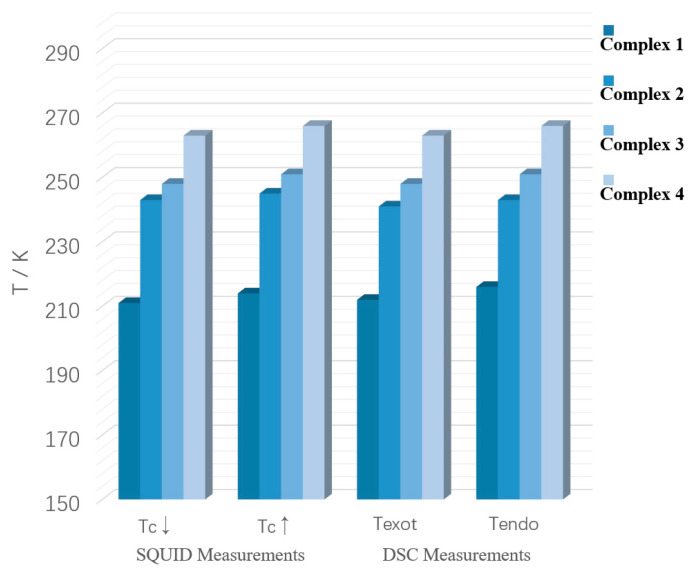
The four specific temperatures T_c_↓, T_c_↑, T_exot,_ and T_endo_ collected from the SQUID and DSC spectra above, corresponding to the spin magnetic transition points of complex **1**, **2**, **3**, **4**. (from the left column to the right in each temperature item).

We sum up the four specific temperatures T_c_↓, T_c_↑, T_exot_, and T_endo_ (turning points), from the data collected from the SQUID and DSC spectra above, corresponding to the spin magnetic transition points of the four complexes with different proportions of polymer ligand and small molecular ligand in Figure 8. We can see the ascending trends of the critical T in each item for the four complexes (four individual columns) for both measurements, no matter whether in cooling or heating processes. That means the more flexible polymer chains attached to the complex or the higher the proportion of polymer chain-modified triazole ligand compared to small ligand in the SCO system, the better malleability it owned and the lower the spin magnetic transition temperature it possessed. In another word, the statistics of T (T_c_↓approximately equals T_exot_, and T_c_↑ approximately equals T_endo_) in both SQUID and DSC measurements having the similar results for all the four complexes proved that the spin magnetic transition points authentically exist and are reliable. So, keeping a balance between processability and SCO magnetic behavior in designing SCO materials should always be in mind.

### 3.5. Crystal Structure Analysis

**Figure 9:**
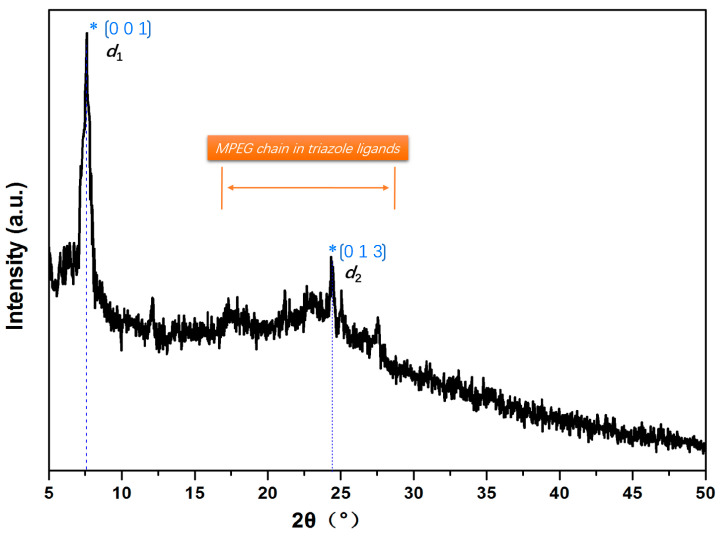
X-ray diffraction profile of the polymeric SCO coordination complex at rt.

Furthermore, we measured the crystal structure of the MPEG-modified Fe (II) complex through XRD, which is shown in Figure 9. We can clearly see the spectrum displays an intensive diffraction peak from 6.5° to 8° (the maximum value of 2θ is 7.6° with (001) plane index, labeled *), which is related to the *d*_1_ (11.67 Å), the spacing of the coordination units between organic ligands triazoles and central Fe^2+^ ions. In other words, the value most possibly suggests an interdigitation of the polymer to give an irregular poly-Fe (II) nuclear chain. For the value of 2θ from 17° to 28.5°, there is a wide diffraction peak attributed to the appearance of amorphous MPEG chains attached to the complex. The reason for the weak intensity and wide pattern of the diffraction peak in XRD the spectrum may be the long flexible MPEG chain on new triazole ligand we synthesized, which further lead to a smaller size of the complex particles and more defective surface. Additionally, the 2θ of the main peak value in this broad range is 24.4° with (013) plane index, which is related to the *d*_2_ (3.65 Å), the distance of adjacent central metal Fe (II) ions in the complex backbone. In addition, we still can deduce *d*_3_, which is the distance between the central Fe^2+^ ions and the nearest nitrogen atom in triazle ligand, using the following equation [32],

(1) d3=d2−1.372cos54°

where the value 1.37 is the distance between atom N_1_ and N_2_ in triazole ligand, and the 54° is the angle of the three atoms N-Fe-Fe linked in one repeat unit. Then, we acquired the distance *d*_3,_ which is 1.94 Å. So, the spectrum of XRD from another point of view provided forceful evidences for the successful synthesis of iron (II) ions coordinated with MPEG-modified triazole ligands polymer complexes. The numbers of repeated units, which are connected or composed through coordination bonds between iron (II) ions and organic triazole ligands in the polymer backbone, are also considerable, by the analysis of the intensity of the diffraction peak.

### 3.6. Computational Study

**Figure 10:**
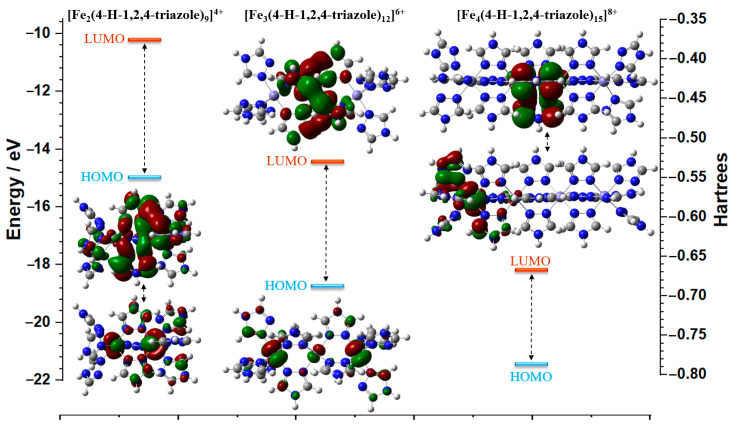
The DFT calculations of HOMO-LUMO energy levels for binuclear (**left**), trinuclear (**middle**), and tetranuclear (**right**)-spin centers of ferrous coordination compound with the same ligand 4-H-1,2,4-triazole.

Theoretical computational study of DFT (Gaussian 09W) was used in Fe (II) SCO polymer complexes for the first time to reveal the relationship between the molecular chain structure and the spin magnetic properties by comparing the HOMO-LUMO energy levels and spin density calculations of fundamental or repeat units in the polymer complex. Gaussian calculations as a conventional DFT simulation to compute the extremely big or long flexible polymer complexes are actually inappropriate, because of too many possible conformations in the macromolecular structures and may lead to inaccurate results or spend too long to complete the calculation results. So, here, we made two groups structural approximations of different four-position substituted [Fe(1,2,4-triazole)_3_]^2+^ derivatives with different length of repeat units in SCO molecules to avoid the drawbacks of DFT. One group is a series of coordination compounds composed by 4-H-1,2,4-triazole ligands in three different lengths of SCO molecules, as shown in Figure 10. They are Fe (II) binuclear spin centers coordinated with nine 4-H-1,2,4-triazole ligands ([Fe_2_(4-H-1,2,4-triazole)_9_]^4+^), Fe (II) trinuclear spin centers with twelve same ligands ([Fe_3_(4-H-1,2,4-triazole)_12_]^6+^), and Fe (II) tetranuclear spin centers with fifteen same ligands ([Fe_4_(4-H-1,2,4-triazole)_15_]^8+^), respectively.

The option of calculation job type we chose was Opt + Freq, and the computational method is Ground State DFT with the theoretical level of Gaussian calculation UB3LYP. The basis set of simulation is by 6-31G(d). The DFT results collected in Figure 10 shows the probability distribution of electron density of each structure, the specific values of HOMO/LUMO energy levels, and the energy gaps Δ*E* between them for each calculated structure. We can clearly see that the HOMO/LUMO energy levels of [Fe_2_(4-H-1,2,4-triazole)_9_]^4+^ are −0.5508/−0.3757 Hartrees. Meanwhile, the HOMO/LUMO energy levels of [Fe_3_(4-H-1,2,4-triazole)_12_]^6+^ and [Fe_4_(4-H-1,2,4-triazole)_15_]^8+^ are −0.6881/−0.5310 Hartrees and −0.7857/−0.6675 Hartrees, respectively. So, the gap Δ*E* between HOMO and LUMO energy levels of the three Fe (II) SCO polymer complexes are 0.1751 Hartrees, 0.1571 Hartrees, and 0.1182 Hartrees, which represent an obvious decline in turn from Fe (II) binuclear spin centers with nine 4-H-triazole ligands to Fe (II) tetranuclear with fifteen 4-H-triazole ligands. The lower the energy gaps Δ*E*, as the repeat unit number of Fe (II) nuclear spin center in the backbone increased, illustrated by closer energy states between ground state and excited states of the SCO polymer complexes, the longer the polymer chain of triazole ligands coordinated Fe (II) possessed. According to the explanation of crystal field theory (CFT), the SCO molecules are capable of transition between two quantum spin states, through thermal excitation. The much narrower energy gaps Δ*E* between ground and excited states make the possibility of transition much greater and much easier between HS (quintet state) and LS (singlet state). So, the DFT Gaussian calculation results corresponding to the series of structural approximations are consistent with CFT, and meanwhile, it also demonstrates that the longer the backbone or the more the repeated units of SCO polymer complexes, the easier the spin magnetic transition in specific thermal excited way. In addition, no obvious rule of variations of the macromolecule dipole moment could be summarized in this derivative series of structural approximations.

**Figure 11:**
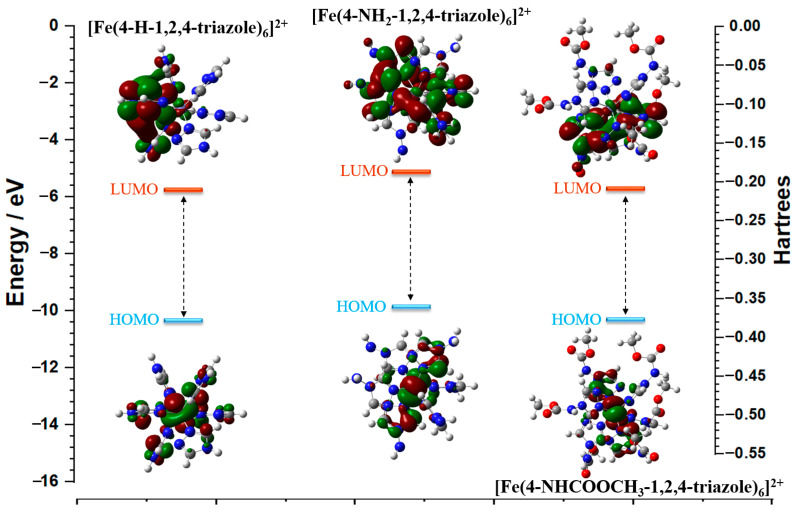
The DFT calculations of HOMO-LUMO energy levels for mononuclear spin center of ferrous sextet coordination compound. (the ligands in the **left** are 4-H-1,2,4-triazole, 4-NH_2_-1,2,4-triazole in the **middle**, and 4-NHCOOCH_3_-1,2,4-triazole in the **right**).

Another group is a series of coordination compounds composed by Fe (II) mononuclear spin center with various four-position substituted triazole derivatives as ligands. The three different four-position substituted organic ligands of SCO molecules are 4-H-1,2,4-triazole, 4-NH_2_-1,2,4-triazole, and 4-NHCOOCH_3_-1,2,4-triazole, respectively, as shown in Figure 11. The collected calculation results: the probability distribution of electron density of each structure, the specific values of HOMO/LUMO energy levels, and the energy gaps Δ*E* between them for each SCO coordination compounds are presented below. The specific values of HOMO/LUMO energy levels of [Fe(4-H-1,2,4-triazole)_6_]^2+^, [Fe(4-NH_2_-1,2,4-triazole)_6_]^2+^, and [Fe(4-NHCOOCH_3_-1,2,4-triazole)_6_]^2+^ are −0.3792/−0.2124 Hartrees, −0.3632/−0.1883 Hartrees, and −0.3781/−0.2100 Hartrees, respectively. So, the deduced gap Δ*E* between HOMO and LUMO energy levels of the three Fe (II) SCO polymer complexes are 0.1668 Hartrees, 0.1749 Hartrees, and 0.1681 Hartrees. No obvious trends of these energy levels or gaps for the variations of the three different four-position substituted groups could be summarized. This indicate that the substituted groups, no matter whether it is weak/strong polarity groups or electric donor/accepter at the edge of the polymer complex backbone, would not regularly bring too many influences on the energy levels of whole complexes. That is to say, the pendant groups on triazole ligands around the ferrous ion would not change the properties of spin magnetic transition so much in the system of crystal field theory. According to the theoretical Gaussian simulations results, the synthesized one-dimensional spin-crossover (SCO) complex {[Fe(MPEG-trz)_3_](BF_4_)_2_} with malleable polymer characteristics still maintain a good performance of spin magnetic transition.

In addition, the SCO molecule dipole moment of [Fe(4-NH_2_-1,2,4-triazole)_6_]^2+^ is an anomalous value and is far less than the dipole moment of other complexes in this series. The anomalous low polarity of [Fe(4-NH_2_-1,2,4-triazole)_6_]^2+^ could be explained by the primary amine, which is used for the four-position substituted group in triazole ligands. Six primary amine groups, which possess the electron donor feature, enwrapped iron dications and weakened the whole molecular polarity to the dipole moment value: 0.208 D. All the corresponding DFT calculated results mentioned above are summarized in Table 3.

**Figure 12:**
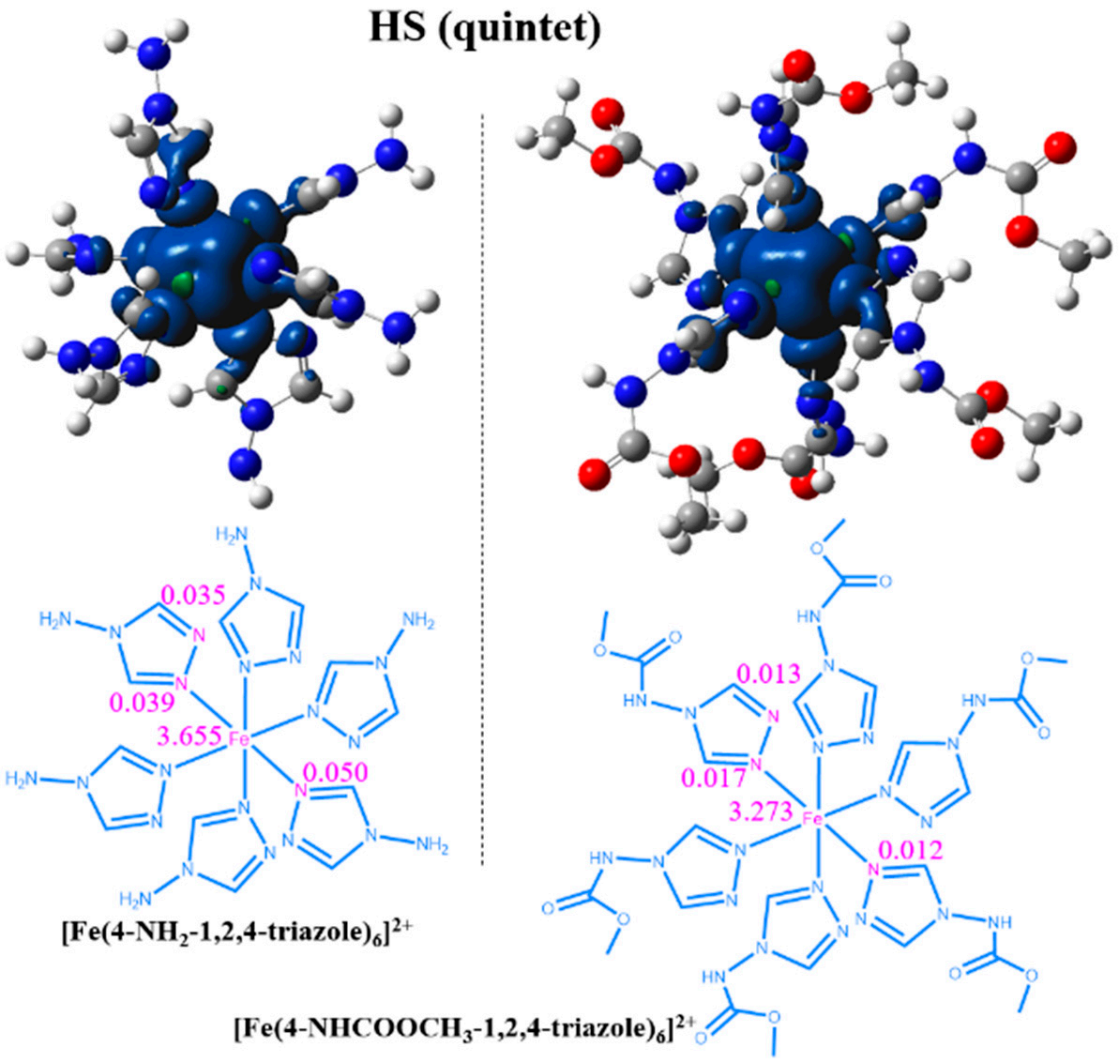
The DFT calculations of spin density distributions and the spin absolute value of partially representative atoms in the mononuclear ferrous sextet coordination compound. (The ligands are 4-NH_2_-1,2,4-triazole in the left, while 4-NHCOOCH_3_-1,2,4-triazole in the right).

In order to explore the relationships between the molecular chain structure, especially the pendant groups on the edge of the polymer chain and the spin density distributions around the spin center ferrous ion (dication radical), DFT computations was used in the SCO systems for the first time to reveal the states of spin clearly in their high spin state (quintet), with the option of spin quantum multiplicity, which changed to quintet. Other information of computational method is the same with above Gaussian calculations. We still use the two, four-position substituted derivatives [Fe(4-NH_2_-1,2,4-triazole)_6_]^2+^ and [Fe(4-NHCOOCH_3_-1,2,4-triazole)_6_]^2+^ mentioned above to simulate the SCO system. From the two upper simulated diagram of spin density distributions, the high spin state’s distributions occupied the main portions of all and concentrated mainly around the spin center ferrous ion, almost till the nitrogen atoms of 1, 2-position of triazole as shown in Figure 12. (We assumed the blue distribution represents the same spin direction, which is considered as the HS state, while the green one is the opposite spin direction.) The atoms in further distance to the spin center in the polymer backbone nearly have no spin density distribution. This conclusion from theoretical Gaussian calculation also illustrated that the different substituted pendants almost will not change the spin transition properties of the whole SCO systems, which gives the long flexible chain methoxy polyethylene glycol (MPEG) modification of SCO materials a strong theoretical support. However, due to the different polarity or electronegativity of substituted pendants, the absolute values of spin density to each spin center atoms may vary. The simulation results reflect that the higher electron donor pendants are more conducive to strengthen the spin absolute values. The specific absolute values of the main spin distributed atoms are clearly shown in the bottom of Figure 12.

## 4. Conclusions

In summary, a novel Fe (II) SCO complex was successfully prepared by the self-assembly of polymer (MPEG)-based ligands triazoles and Fe(BF_4_)_2_•6H_2_O. According to the discussions above, the MPEG-functionalized polymer ligands exhibit big differences in spin magnetic properties or transitions (e.g., the critical temperature of spin transition or the width of magnetic hysteresis loop and so on) and also the material processability compared to the small triazole derivative molecules attached to central metal ions. Both SQUID (the temperature range: 10~300 K) and DSC (the temperature range: 195~290 K) results indicated an evident spin crossover performance and slender range of hysteresis loop. These results illustrate that a specific SCO complex with excellent malleability was realized. These results signify that the preparation of a soft matter such as a polymer film possessing an excellent spin magnetic switching property is definitely possible. In addition, the crystal structure of the modified SCO complex was resolved using XRD analysis. The DFT computations displayed the series of HOMO-LUMO energy levels of [Fe(1,2,4-triazole)_3_]^2+^ derivatives in different sizes and concluded the variation trend of the energy gaps Δ*E* with the change of macromolecular length and also the relationships between the structures and the transition of spin states. The theoretical Gaussian simulation revealed the rule that the spin density distributions on the macromolecules were affected by the substituted groups of ligand triazoles. Furthermore, various molecular weight MPEG-functionalized SCO complexes could be facilely introduced into the spin crossover system, which are currently under progress, for investigating the mechanism of spin magnetic transition and also malleability.

## Figures and Tables

**Table 1:** The magnetic properties of spin transitions at T_c_↓ and T_c_↑ for complex **1**, **2**, **3**, **4**.

SCO Complex	T_c_↓ *^a^* (K)	T_c_↑ *^b^* (K)
Complex **1**	211	214
Complex **2**	243	245
Complex **3**	248	251
Complex **4**	263	266

*^a^* The critical temperature, at which one half of the SCO behavior is completed during cooling process. *^b^* The critical temperature, at which one-half of the SCO behavior is completed during heating process.

**Table 2:** The temperature at exothermic and endothermic peaks of complex **1**, **2**, **3**, **4**.

SCO Complex	T_exot_ *^a^* (K)	T_endo_^*b*^ (K)
Complex **1**	212	216
Complex **2**	241	243
Complex **3**	248	251
Complex **4**	263	266

*^a^* The critical turning temperature at exothermic peak, during cooling process. *^b^* The critical turning temperature at endothermic peak, during heating process.

**Table 3:** The orbital energy levels and the energy gaps between HOMO and LUMO of six simulated Fe (II) triazoles coordination structures.

Fe (II) Triazoles Coordination	LUMO/Hartrees	HOMO/Hartrees	Energy Gap ΔE/Hartrees	Dipole Moment/D
[Fe_2_(4-H-1,2,4-triazole)_9_]^4+^	−0.3757	−0.5508	0.1751	3.131447
[Fe_3_(4-H-1,2,4-triazole)_12_]^6+^	−0.5310	−0.6881	0.1571	4.758551
[Fe_4_(4-H-1,2,4-triazole)_15_]^8+^	−0.6675	−0.7857	0.1182	3.924752
[Fe(4-H-1,2,4-triazole)_6_]^2+^	−0.2124	−0.3792	0.1668	3.754252
[Fe(4-NH_2_-1,2,4-triazole)_6_]^2+^	−0.1883	−0.3632	0.1749	0.207855
[Fe(4-NHCOOCH_3_-1,2,4-triazole)_6_]^2+^	−0.2100	−0.3781	0.1681	2.146118

## Data Availability

Data are available upon request.
